# Stroke Subtype as a Determinant of Mortality in Adult Patients on Extracorporeal Membrane Oxygenation

**DOI:** 10.3390/jcm15124790

**Published:** 2026-06-20

**Authors:** Amir Mahdi Ghafarian, Ali Samani, Jawad Saad, Mohammad Ghafarian, Muaaz Wajahath, Sarah Foster, Seungwon Lim, Aliyah Sutton, Faddi G. Saleh Velez, Denise Battaglini, Andrea Loggini

**Affiliations:** 1 School of Medicine, International University of the Health Sciences, Basseterre, St. Kitts and Nevis, 706 W Main Street, Carbondale, IL 62901, USA; 2 School of Medicine, Toronto Metropolitan University, Toronto, ON L6T 2T9, Canada; asamani@torontomu.ca; 3 School of Medicine, Wayne State University, Detroit, MI 48201, USA; jawads@wayne.edu; 4 Department of Family & Community Medicine, University of Toronto, Toronto, ON M5S 1A1, Canada; ghafarianm@gmail.com; 5 College of Human Medicine, Michigan State University, East Lansing, MI 48824, USA; wajahath@msu.edu; 6 School of Medicine, Southern Illinois University, Carbondale, IL 62901, USA; sfoster99@siumed.edu (S.F.); slim36@siumed.edu (S.L.); andrea.loggini@sih.net (A.L.); 7 School of Medicine, St. George’s University, True Blue, St. George, Grenada; asutton1@sgu.edu; 8 Brain Stimulation and Neurorehabilitation Laboratory, Department of Neurology, University of Oklahoma Health Sciences Center, Oklahoma City, OK 73117, USA; faddi-salehvelez@ou.edu; 9 Anesthesia and Intensive Care, IRCCS Azienda Ospedaliera Metropolitana, 16132 Genova, Italy; denise.battaglini@unige.it; 10 Department of Surgical Sciences and Integrated Diagnostics, University of Genoa, 16126 Genoa, Italy; 11 Brain and Spine Institute, Southern Illinois Healthcare, Carbondale, IL 62901, USA

**Keywords:** stroke, extracorporeal membrane oxygenation, ECMO, intensive care unit, hemorrhagic stroke, ischemic stroke, mortality, propensity score matching

## Abstract

**Background:** Stroke significantly increases morbidity and mortality in patients receiving extracorporeal membrane oxygenation (ECMO). This study evaluates the prognostic impact of stroke subtypes, acute ischemic stroke (AIS) and hemorrhagic stroke (HS), and neurologic injury severity in a contemporary adult population. **Methods:** We conducted a retrospective cohort study using the TriNetX federated electronic health record network, including adult patients who underwent ECMO between 1 October 2015 and 31 December 2025. Stroke was defined as a first-instance diagnosis of AIS, HS, or unspecified cerebrovascular event occurring within 24 h of ECMO cannulation during the index hospitalization. Propensity score matching (1:1 nearest neighbor) was performed to balance baseline demographics, comorbidities, anticoagulant use, and ECMO modality between the stroke and non-stroke cohorts. Primary outcomes included all-cause mortality at 30 days, 90 days, and 1 year. Secondary outcomes included cardiac arrest, seizures, palliative care utilization, and hospital readmission. Kaplan–Meier survival analysis and multivariable Cox proportional hazards modeling were performed. **Results:** Among 18,981 ECMO patients, 1481 (7.8%) developed a stroke within 24 h of ECMO cannulation, including 814 AIS (54.9%), 454 HS (30.6%), and 213 unspecified cerebrovascular events (14.4%). After propensity score matching, stroke was associated with significantly higher all-cause mortality at 30 days (RR 1.16), 90 days (RR 1.18), and 1 year (RR 1.18), all *p* < 0.05. Stroke was also associated with higher rates of cardiac arrest, seizures, hospital readmission, and palliative care utilization (all *p* < 0.001). AIS was associated with significantly lower mortality than HS at 30 days, 90 days, and 1 year (all *p* < 0.0001). In multivariable Cox regression, only HS was independently associated with increased 30-day mortality compared with no stroke. Markers of neurologic injury severity, including cerebral edema, brain compression, and coma, were among the strongest independent predictors of mortality. **Conclusions:** Stroke occurring early after ECMO cannulation is associated with substantially worse short- and long-term survival, with hemorrhagic subtype and markers of neurologic injury severity driving the strongest prognostic signals. These findings support early stroke recognition and subtype-informed prognostic discussions in ECMO patients.

## 1. Introduction

Extracorporeal membrane oxygenation (ECMO) has become a crucial life-support modality for patients with refractory cardiac and respiratory failure, with expanding indications and improving survival across critical care settings. While the utilization of both venoarterial (VA) and venovenous (VV) configurations has expanded significantly over the last decade, the survival benefit of ECMO remains tempered by a high incidence of non-cardiac complications [1,2,3].

Stroke is among the most feared neurologic complications during ECMO support. Reported incidence varies widely across studies and ECMO configurations, ranging from 1% to 10% for ischemic stroke and 2% to 8% for intracranial hemorrhage, reflecting differences in patient selection, cannulation strategy, anticoagulation protocols, and case ascertainment methods [4,5,6]. The pathophysiology of cerebrovascular injury during ECMO is multifactorial, encompassing thromboembolism related to the extracorporeal circuit, cerebral hypoperfusion, anticoagulation-related hemorrhagic risk, and underlying cardiovascular pathology [7,8]. Large registry analyses from the Extracorporeal Life Support Organization (ELSO) have characterized the epidemiology of stroke in both VA-ECMO and VV-ECMO populations and have established that both ischemic and hemorrhagic events are associated with substantially higher in-hospital and 90-day mortality compared with ECMO patients without neurologic complications [4,5,9]. Hemorrhagic stroke, in particular, has consistently been shown to carry a worse prognostic profile than ischemic stroke, with in-hospital mortality approaching 86% in some registry series, reflecting the catastrophic consequences of intracranial hemorrhage in systemically anticoagulated and hemodynamically unstable patients [4].

Despite this established epidemiological foundation, important gaps persist. First, most large-scale analyses of stroke in ECMO have relied on voluntary registry data from the ELSO network, which, while invaluable, is subject to potential selection bias, variability in complication reporting practices across contributing centers, and limited capture of downstream outcomes such as hospital readmission and palliative care utilization [2,4,5]. Second, the majority of ELSO-based analytic studies use datasets ending in 2021, while detailed post-2021 outcome analyses in the analytic literature remain limited [2,3]. Third, while seizures in ECMO patients have been reported at approximately 1–2% prevalence in registry analyses, their prognostic significance within the stroke subpopulation, as distinct from broader acute brain injury composites, has not been well characterized [10,11]. Finally, outcomes including palliative care utilization and hospital readmission following stroke in ECMO patients have received minimal attention in the existing literature, despite their direct relevance to resource allocation, prognostic discussions, and goals-of-care planning [12].

To address these gaps, this study used the TriNetX federated electronic health record (EHR) network, a real-world data environment independent of voluntary registry reporting, to examine stroke-associated outcomes in a large, contemporary cohort of adult ECMO patients from 2015 to 2025. Rather than seeking to establish causal mechanisms, this analysis aims to: (1) characterize the incidence and clinical profile of stroke in a broad, real-world EHR-based ECMO cohort; (2) confirm and extend prior registry findings on stroke-associated mortality using an independent data source with propensity score matching to adjust for baseline illness severity; (3) compare short- and long-term outcomes by stroke subtype; and (4) examine the association between markers of neurologic injury severity and mortality in multivariable modeling. By replicating and extending prior ELSO registry findings in a distinct data environment and by characterizing outcomes that have received limited prior attention, including palliative care utilization and readmission, this analysis contributes confirmatory and complementary evidence to the existing ECMO stroke literature.

## 2. Materials and Methods

### 2.1. Data Source and Study Design

A retrospective cohort study was conducted using the TriNetX federated electronic health record (EHR) research network(TriNetX, LLC, Cambridge, MA, USA), a platform that aggregates de-identified, longitudinal data from participating healthcare organizations across the United States, including demographics, diagnoses, procedures, medications, and outcomes derived from real-world clinical encounters. TriNetX has been extensively validated and applied in peer-reviewed observational research across a broad range of clinical domains, including neurologic and cardiovascular outcomes research [1,2,3]. The platform employs standardized data quality protocols across contributing sites and restricts analytical access to aggregate, de-identified data, precluding patient-level identification. Because all data were de-identified and accessed through a federated analytics environment without direct patient contact, this study was determined to be exempt from institutional review board approval in accordance with 45 CFR 46.104(d). This study is reported in accordance with the Strengthening the Reporting of Observational Studies in Epidemiology (STROBE) guidelines [13].

### 2.2. Study Population

Adult patients aged 18 years or older who were hospitalized in the United States and underwent ECMO between 1 October 2015 and 31 December 2025 were identified using ICD-10-PCS procedure codes. The study period was anchored to 1 October 2015, coinciding with the federally mandated transition from ICD-9 to ICD-10 coding in United States hospitals, ensuring code consistency throughout the observation window [14]. ECMO was defined by the presence of any of the following ICD-10-PCS procedure codes: peripheral venoarterial ECMO (5A1522G), peripheral venoarterial ECMO intraoperative (5A15A2G), peripheral venovenous ECMO (5A1522H), or peripheral venovenous ECMO intraoperative (5A15A2H), consistent with prior administrative database studies identifying ECMO cohorts using ICD-10-PCS procedural coding [15,16].

The index date was defined as the first documented ECMO cannulation during the hospitalization. To establish a clear and biologically plausible temporal relationship between ECMO initiation and cerebrovascular injury, stroke was defined as a new ICD-10 diagnosis code recorded within 24 h following the index cannulation date and during the same index hospitalization. This window was selected to capture the peri-cannulation neurologic injury period, encompassing the hemodynamic, embolic, and anticoagulation-related mechanisms most directly attributable to circuit initiation; although cerebrovascular events, particularly hemorrhagic events arising against the background of prolonged anticoagulation, may also occur later during ECMO support or weaning, restricting ascertainment to this interval was a deliberate decision to preserve a defensible temporal link between cannulation and the index neurologic event.

Stroke events were categorized as acute ischemic stroke (AIS; I63.x), hemorrhagic stroke (HS; I60.x–I62.x), or unspecified cerebrovascular event (I64), as detailed in Supplementary Table S1. ICD-10 codes I63.x for AIS and I60.x–I62.x for HS have demonstrated high specificity and positive predictive value for stroke case ascertainment in administrative data; in validation studies these more specific subtype codes have shown positive predictive values of approximately 82% for ischemic stroke, 89% for intracerebral hemorrhage, and 93% for subarachnoid hemorrhage, with specificity of 95% or greater, supporting their use in large observational studies [17,18]. The unspecified stroke group (I64) was retained in the overall stroke versus no-stroke comparison but excluded from subtype-specific analyses given the clinical heterogeneity of this category and its recognized limitations as a research code [19]. Patients without any stroke diagnosis during the defined 24 h post-cannulation window were used as the comparative group.

### 2.3. Covariates

Baseline covariates were ascertained from diagnostic and procedural codes recorded prior to the index date of ECMO cannulation, as defined within the TriNetX platform’s temporal framework, which explicitly separates pre-index from post-index events. This architecture ensures that all covariates represent pre-existing conditions rather than complications arising during ECMO support. Baseline demographic variables included age, sex, race, and ethnicity. Clinical covariates were selected based on prior ECMO and stroke literature and included the following conditions, each identified by the ICD-10 code(s) noted: heart failure (I50), cardiomyopathy (I42), atrial fibrillation and flutter (I48), paroxysmal tachycardia (I47), other cardiac arrhythmias (I49), acute myocardial infarction (I21), subsequent ST-elevation and non-ST-elevation myocardial infarction (I22), acute myocarditis (I40), myocarditis unspecified (I51.4), coronary artery disease (I25), respiratory failure (J96), shock (R57), sepsis (A41), acute kidney injury (N17), coma (R40.20–R40.24), anoxic brain damage (G93.1), prior neurologic injury, pulmonary embolism (I26), other venous embolism and thrombosis (I82), gastrointestinal hemorrhage (K92.2), critical illness myopathy (G72.81), and critical illness polyneuropathy (G62.81) [4,5]. Anticoagulant medication use was captured using the TriNetX drug classification code BL110, which encompasses all anticoagulant therapeutic classes as a group. ECMO modality was captured using the procedure codes described above, with VA-ECMO defined by codes 5A1522G and 5A15A2G and VV-ECMO defined by codes 5A1522H and 5A15A2H. All ICD-10 code definitions for covariates and outcomes are provided in Table S1.

### 2.4. Outcomes

The primary outcome was all-cause mortality at 30 days, 90 days, and 1 year following ECMO cannulation. Secondary outcomes measured from the index date within predefined follow-up windows included cardiac arrest (I46), epilepsy or recurrent seizures (G40, R56.9), palliative care encounter (Z51.5), and hospital readmission (SNOMED 32485007). Palliative care utilization has been characterized as a clinically meaningful outcome in critically ill cardiovascular and neurologic populations, and its capture in administrative data has been applied in prior large-scale observational studies [20]. Cerebral edema (G93.6), cerebral compression and herniation (G93.5), and coma (R40.20–R40.24) were included as covariates in the multivariable Cox regression model given their established association with mortality in acute neurologic populations.

### 2.5. Propensity Score Matching

To reduce confounding attributable to measured baseline differences in illness severity and comorbidity burden, propensity score matching (PSM) was performed between ECMO patients who developed stroke within 24 h of cannulation and those who did not. The propensity score was estimated using a logistic regression model incorporating demographics, clinical comorbidities, anticoagulant use (BL110), and ECMO modality (5A1522G, 5A15A2G, 5A1522H, 5A15A2H) as covariates. A 1:1 nearest-neighbor matching algorithm without replacement was applied within a caliper of 0.1 standard deviations of the logit of the propensity score. Nearest-neighbor caliper matching has been shown to minimize estimation bias while maintaining sample efficiency in observational studies, and the 0.1 standard deviation caliper has been widely applied in published propensity-matched analyses [21,22]. Covariate balance between matched cohorts was assessed using standardized mean differences (SMD), with values less than 0.1 indicating adequate balance, consistent with established methodological thresholds for propensity score-matched studies [23]. A separate PSM analysis was performed within the stroke cohort to compare patients with AIS (I63.x) against those with HS (I60.x–I62.x), using the same matching procedure.

### 2.6. Statistical Analysis

A formal sample size calculation was not performed, as this was a retrospective observational study in which the sample size was determined by the number of eligible patients within the database during the study period. Categorical variables were summarized as counts and percentages; continuous variables were presented as mean and standard deviation. Between-group comparisons of outcomes in propensity-matched cohorts were performed using risk differences, risk ratios (RR), and odds ratios (OR) with 95% confidence intervals. Time-to-event analyses for all-cause mortality were conducted using Kaplan–Meier survival estimation with log-rank testing to assess between-group differences. Multivariable Cox proportional hazards regression was performed to identify variables independently associated with 30-day mortality, adjusting for demographic characteristics, comorbidities, markers of neurologic injury severity, and ECMO modality. All statistical analyses were conducted within the TriNetX platform using its validated built-in analytical tools. Statistical significance was defined as a two-sided *p*-value less than 0.05. Since this study included all eligible patients, statistical power was determined by the size of the available cohort rather than by a prespecified target; the large matched sample (1480 pairs) produced narrow confidence intervals around the primary mortality estimates, indicating adequate precision to detect clinically meaningful differences, and post hoc power was not computed, as power derived from observed effect estimates is uninformative beyond the reported confidence intervals. Given the number of secondary outcomes and time points examined, no formal correction for multiple comparisons was applied; the principal mortality comparisons were significant at *p* ≤ 0.0006 and would remain significant under conventional correction, whereas the small number of secondary findings with borderline *p*-values are interpreted as exploratory and hypothesis-generating rather than confirmatory.

## 3. Results

### 3.1. Study Population

**Figure 1:**
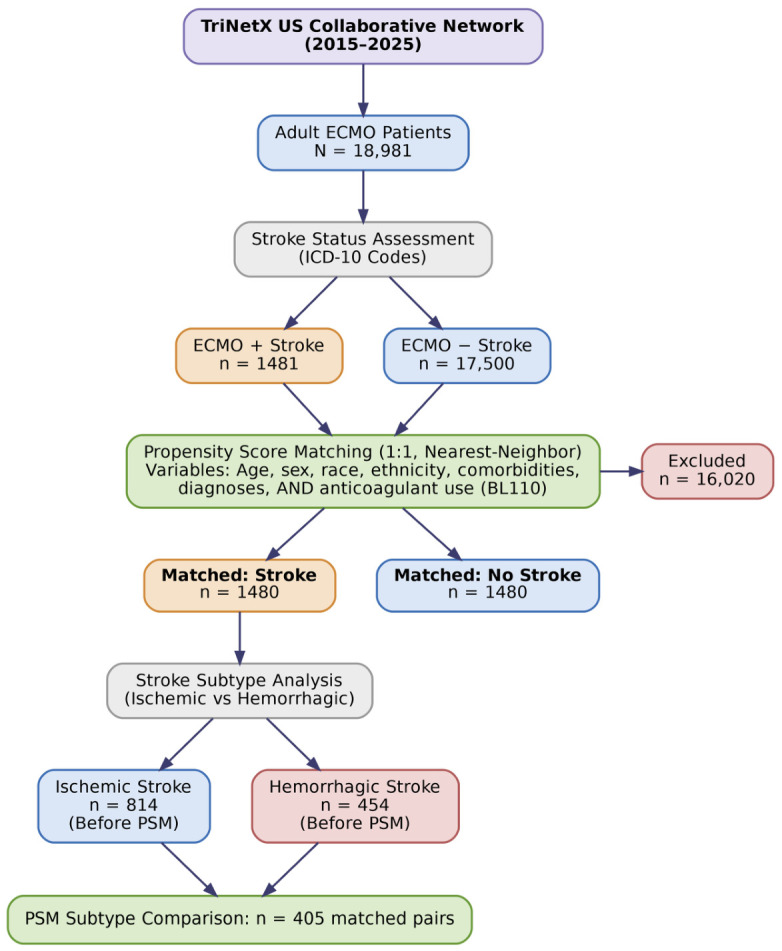
Study design and patient flow diagram. Adult patients undergoing ECMO between 1 October 2015 and 31 December 2025 were identified from the TriNetX federated EHR network. Stroke was defined as a new ICD-10 diagnosis within 24 h after ECMO cannulation and during the index hospitalization. Patients were stratified into stroke and no-stroke cohorts, then matched 1:1 by propensity score; a parallel subtype matching procedure compared ischemic (I63.x) and hemorrhagic (I60.x–I62.x) strokes. The unspecified cerebrovascular event group (I64) was excluded from subtype-specific analyses.

Stroke occurred within 24 h of ECMO cannulation in 1481 patients (7.8%), of whom 814 (54.9%) experienced AIS, 454 (30.6%) HS, and 213 (14.4%) unspecified cerebrovascular events (ICD-10 I64). The unspecified group was retained in the stroke versus no-stroke comparison but excluded from subtype-specific analyses (Figure 1; Table S2).

### 3.2. Baseline Characteristics and Propensity Score Matching

Prior to matching, substantial baseline differences were observed between cohorts, with SMDs exceeding 0.1 for respiratory failure, acute kidney injury, shock, sepsis, heart failure, and atrial fibrillation and flutter (Table 1). Following 1:1 nearest-neighbor propensity score matching, 1480 matched pairs were identified, with all post-match SMDs below 0.1, including for ECMO modality (Table 1).

### 3.3. Stroke and Clinical Outcomes

**Figure 2:**
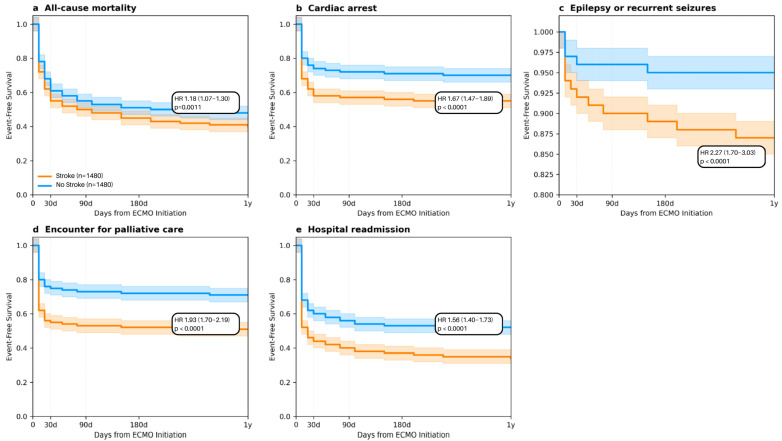
Kaplan–Meier survival curves: stroke vs. no stroke. Kaplan–Meier curves illustrating event-free survival for (**a**) all-cause mortality, (**b**) cardiac arrest, (**c**) epilepsy or recurrent seizures, (**d**) palliative care utilization, and (**e**) hospital readmission in propensity score-matched ECMO patients with and without stroke (n = 1480 per group). Hazard ratios (HRs) with 95% confidence intervals displayed on the plots were derived from Cox proportional hazards regression models.

Stroke was associated with significantly higher all-cause mortality compared with matched controls at 30 days (45.4% vs. 39.2%; RR 1.16, 95% CI 1.07–1.26; *p* = 0.0006), 90 days (52.6% vs. 44.7%; RR 1.18, 95% CI 1.09–1.27; *p* < 0.0001), and 1 year (57.0% vs. 48.2%; RR 1.18, 95% CI 1.11–1.27; *p* < 0.0001) (Supplementary Table S3). Time-to-event analysis confirmed elevated mortality hazard (HR 1.18, 95% CI 1.07–1.30; log-rank *p* < 0.001) (Figure 2). Stroke was also associated with higher rates of cardiac arrest (41.6% vs. 26.5%; RR 1.57), seizures (8.2% vs. 3.9%; RR 2.12), hospital readmission (56.1% vs. 39.9%; RR 1.41), and palliative care encounters (44.9% vs. 24.9%; RR 1.80), all *p* < 0.0001, with associations persisting through 1 year (Supplementary Table S3).

### 3.4. Stroke Subtype and Clinical Outcomes

HS was associated with substantially worse mortality than AIS at all time points: 30 days (56.3% vs. 36.1%; RR 0.64, favoring AIS; *p* < 0.0001), 90 days (64.4% vs. 43.2%; RR 0.67; *p* < 0.0001), and 1 year (67.7% vs. 49.1%; RR 0.73; *p* < 0.0001) (Supplementary Table S4). Secondary outcomes were largely similar between subtypes. Cardiac arrest at 90 days and hospital readmission at 1 year were nominally higher among AIS patients but should be interpreted cautiously, given borderline *p*-values (*p* = 0.047 and *p* = 0.048) and survival bias favoring the AIS group.

Secondary outcomes were largely similar between stroke subtypes. Rates of epilepsy or recurrent seizures and palliative care encounters did not differ significantly between AIS and HS at any time point (Supplementary Table S4). Cardiac arrest rates were comparable at 30 days and 1 year, with a nominally significant increase observed at 90 days among AIS patients (RR 1.18, 95% CI 1.00–1.38; *p* = 0.047), though this finding should be interpreted cautiously given its marginal statistical significance. Hospital readmission rates were similar at 30 and 90 days but were modestly higher among AIS patients at 1 year (59.0% vs. 52.1%; RR 1.13, 95% CI 1.00–1.28; *p* = 0.048), a finding that likewise warrants cautious interpretation given the borderline *p*-value and the higher overall survival in the AIS group, which creates greater opportunity for readmission.

### 3.5. Predictors of Mortality Among Stroke Patients

In multivariable Cox modeling, AIS was not independently associated with 30-day mortality compared with no stroke (HR 0.99, 95% CI 0.87–1.12; *p* = 0.81), while HS was strongly associated (HR 1.37, 95% CI 1.19–1.58; *p* < 0.0001). In direct subtype comparison, AIS carried substantially lower mortality risk than HS (HR 0.69, 95% CI 0.57–0.82; *p* < 0.0001) (Table 2, Supplementary Tables S5–S7).

Markers of neurologic injury severity showed the strongest associations with mortality across all models: cerebral edema (HR 2.60, 95% CI 2.16–3.12), brain compression (HR 2.41, 95% CI 1.89–3.06), and coma (HR 1.21, 95% CI 1.06–1.38), all *p* < 0.005. These associations likely reflect the prognostic burden of severe cerebrovascular injury rather than discrete causal pathways. Shock and acute kidney injury were consistently associated with increased mortality across all three models (all *p* < 0.001). Hypertension, atrial fibrillation, cardiomyopathy, and other arrhythmias demonstrated hazard ratios below 1.0 across all models. Seizure disorder was associated with increased mortality only in the direct subtype comparison model (HR 1.38, 95% CI 1.004–1.91; *p* = 0.047).

## 4. Discussion

In this large-scale retrospective cohort study of 18,981 ECMO patients, we identified three key findings regarding cerebrovascular complications during ECMO support. First, validating the existing registry literature, stroke affects approximately 8% of adult ECMO patients and persists as a strong driver of short-term mortality, with a 30-day mortality of 45.4% in stroke patients versus 39.2% in matched non-stroke patients (RR 1.16, 95% CI 1.07–1.26; *p* = 0.0006). Furthermore, after propensity score matching, ECMO patients with stroke demonstrated significantly higher utilization of palliative care compared with matched ECMO patients without stroke at 30 days (44.9% vs. 24.9%; RR 1.80, 95% CI 1.62–2.01), 90 days, and 1 year (all *p* < 0.001). These findings are consistent with prior reports demonstrating the substantial neurologic morbidity and prognostic impact of cerebrovascular complications during extracorporeal life support [24].

To our knowledge, this represents one of the largest studies evaluating stroke outcomes in ECMO patients. In our cohort, approximately 8.1% of patients developed a cerebrovascular event during hospitalization, with AIS accounting for 56% of cases and HS for 31.8% of cases. These findings are consistent with prior reports, which have described stroke incidence ranging from 3% to 10% in the ECMO population [4]. Previous smaller cohort studies have similarly demonstrated that AIS is more frequently observed than HS, likely reflecting the combined effects of thromboembolism related to the extracorporeal circuit, systemic hypoperfusion, and cardiovascular pathologies [25].

Several prior studies have also reported high mortality in ECMO patients who develop stroke. For example, both single-center series and large registry analyses demonstrate markedly elevated mortality once stroke occurs during ECMO support, with reported in-hospital mortality commonly approaching ~70% and often higher for hemorrhagic than ischemic stroke [26,27]. Our findings not only confirm these observations in a larger and more diverse population but also extend prior work by rigorously adjusting for baseline illness severity and directly comparing outcomes by stroke subtype (all post-match SMDs < 0.1).

### 4.1. Differential Mortality by Stroke Subtype

The substantially higher mortality associated with HS compared with AIS, with 30-day mortality exceeding 56% and HS the only independently associated subtype in multivariable modeling, is consistent with prior ECMO-specific evidence. Although our 30-day hemorrhagic-stroke mortality of 56.3% is lower than the in-hospital mortality approaching 86% reported in some ELSO registry series [4], this difference is expected and reflects methodological distinctions rather than a discrepancy in the underlying phenomenon. The ELSO figure represents in-hospital mortality among voluntarily reported registry events, whereas our estimate is a fixed 30-day, propensity-matched mortality derived from a broad, contemporary real-world EHR cohort; moreover, our 24 h ascertainment window captures early peri-cannulation hemorrhagic events specifically, and the matched comparator design, larger and less selected population, and more recent treatment era would each be expected to yield lower absolute mortality than older, selected registry subgroups. Le Guennec et al. found that intracranial bleeding, but not ischemic stroke, was independently associated with mortality in 878 VA-ECMO patients, with hemorrhagic events occurring earlier after cannulation and associated with low platelet count and central cannulation [8]. Fletcher-Sandersjöö et al. reported 30-day mortality of 74% among 65 adult ECMO patients with intracranial hemorrhage, with surgical treatment attempted in only 5 patients, reflecting the prohibitive operative bleeding risk in fully anticoagulated patients [28]. Prinz et al. corroborated this, finding that early coagulation correction was achievable in only 63% of cases and was the key determinant of intracranial hemorrhage-related survival [29]. The ELSO neurologic monitoring consensus guidelines accordingly recommend individualized anticoagulation management given the competing hemorrhagic and thrombotic demands inherent to ECMO support [11].

In the broader neurocritical care literature, intracranial hemorrhage carries a consistently higher early mortality than ischemic stroke, driven by rapid hematoma expansion, mass effect, and the limited capacity for surgical intervention in anticoagulated patients [26,30]. In ECMO patients, systemic anticoagulation further amplifies this risk: Mansour et al., in a nationwide French COVID-19 ECMO registry, reported intracranial hemorrhage in 8% of 620 patients with an adjusted odds ratio of 13.5 for in-hospital mortality, the strongest mortality association among all bleeding types [31].

### 4.2. Neurologic Severity Markers and Seizures

Cerebral edema, brain compression, and coma showed the strongest associations with mortality across all Cox models. These variables are best interpreted as ICD-10 proxies for severe neurologic injury rather than as independent causal drivers, a distinction with direct implications for how these findings are communicated clinically. This interpretation is consistent with Peluso et al., who found that suppressed EEG background, a neurophysiological correlate of severe cerebrovascular injury, was independently associated with unfavorable 3-month outcome in 139 EEG-monitored adult ECMO patients (OR 10.08, 95% CI 1.24–82.20) [32].

The more than twofold higher coded seizure rate in stroke patients (8.2% vs. 3.9%) almost certainly underestimates the true burden. In sedated, mechanically ventilated ECMO patients, seizures are predominantly nonconvulsive and require continuous EEG monitoring for detection. Kohne et al. identified seizure diagnoses in 20% of a large pediatric ECMO registry cohort, rates far exceeding those detectable by clinical observation in sedated patients [33]. The ELSO consensus guidelines recommend targeted or continuous EEG monitoring for ECMO patients with new neurologic symptoms, a recommendation that the present coding-based findings cannot adequately evaluate but indirectly support through the substantially higher seizure burden and worse outcomes observed in the stroke cohort [11]. Several chronic cardiac conditions, including hypertension, atrial fibrillation, cardiomyopathy, and other arrhythmias, were associated with hazard ratios below 1.0 in the multivariable models. These counterintuitive associations should not be interpreted causally. Patients with previously recognized cardiac disease likely receive earlier diagnosis, guideline-directed therapy, and more intensive monitoring. Furthermore, index-event (collider) bias, inherent to a cohort selected for a severe acute event such as ECMO cannulation, can induce apparently protective associations.

### 4.3. Palliative Care Utilization and Hospital Readmission

The finding that nearly 45% of ECMO patients with stroke accessed palliative care within 30 days, nearly double the rate in matched controls, has not been previously quantified in the ECMO stroke literature. The 2024 American Heart Association (AHA) scientific statement on palliative and end-of-life care in critical cardiovascular illness and the AHA stroke palliative care scientific statement both identify early goals-of-care integration as a clinical imperative for high-acuity neurologic populations [34,35]. These data provide support for that recommendation and suggest that structured palliative care consultation, particularly in patients with hemorrhagic stroke or markers of severe neurologic injury, should be initiated at the time of stroke diagnosis rather than deferred until clinical deterioration.

Readmission rates were substantially higher in stroke patients at all time points (56.1% at 30 days vs. 39.9% in controls), far exceeding rates reported in community stroke populations. Zhou et al. reported 30-day and 1-year readmission rates of 9.7% and 30.5%, respectively, following acute ischemic stroke in a national US cohort [36]. Sennfält et al., analyzing 10,092 Swedish stroke registry patients, found that 43.7% were readmitted within 12 months [37]. The substantially higher rates in the present study reflect the compounding vulnerabilities of ECMO survivors, including critical illness severity, multiorgan dysfunction, prolonged antithrombotic dependence, and hemodynamic fragility. The modestly higher 1-year readmission rate among AIS versus HS survivors reflects survival bias: AIS patients surviving to the point of potential readmission constitute a prognostically favorable subgroup with a greater opportunity for subsequent hospitalization events.

Finally, because ischemic stroke subtypes differ substantially in their risk-factor profiles, severity, and prognosis, an important direction for future research will be to evaluate subtype-specific outcomes, distinguishing cardioembolic, atherothrombotic, lacunar, and other etiologies, among ECMO patients, an analysis that will require data sources with imaging-level and etiologic granularity beyond that available in administrative records [38]. More broadly, prospective and multicenter studies that capture variables unavailable in administrative data, including anticoagulation intensity and monitoring, circuit characteristics and duration, cannulation strategy, and validated illness-severity scores, will be essential to clarify the mechanisms linking stroke subtype to mortality during ECMO. Future work should also examine whether imaging- and electroencephalography-adjudicated ascertainment improves detection of cerebrovascular and seizure events in this population, and whether early, subtype-informed neuromonitoring and goals-of-care pathways meaningfully alter outcomes.

### 4.4. Limitations

This study has several limitations. First, its retrospective observational design precludes causal inference, and all findings should be interpreted as associations. Despite propensity score matching on a comprehensive set of measured baseline covariates, residual confounding from unmeasured variables remains a significant concern. Key determinants of both stroke risk and mortality that are unavailable in TriNetX include anticoagulation intensity and therapeutic monitoring, vasopressor burden, hemodynamic trajectory, ECMO circuit duration, circuit-related complications, and adjunct mechanical circulatory support. Validated illness severity scores, including SOFA, SAVE, and RESP, were similarly unavailable, limiting the ability to fully account for baseline critical illness severity. As a result, the possibility that the observed associations partly reflect differences in underlying illness severity rather than stroke itself cannot be fully excluded, and a prospective study incorporating these granular physiologic and ECMO-specific variables would be needed to more definitively address residual confounding. Additionally, extracorporeal cardiopulmonary resuscitation (ECPR) cannot be separately identified from conventional VA-ECMO using ICD-10-PCS procedure codes, and its inclusion within the VA-ECMO population may overestimate stroke-associated neurologic morbidity, given the pre-existing anoxic brain injury burden inherent to cardiac arrest preceding ECPR cannulation. Similarly, cannulation strategy, intra-course circuit changes, and dynamic anticoagulation adjustments, all of which may influence cerebrovascular risk, could not be ascertained from the available procedural and diagnostic codes, and modality-specific findings should therefore be interpreted with these constraints in mind.

Second, stroke subtype classification relies entirely on ICD-10 diagnostic codes without imaging adjudication, and a proportion of events were coded as unspecified cerebrovascular events and could not be further characterized. Because unspecified cerebrovascular events (I64) were included only in the overall stroke versus no-stroke comparison and excluded from all subtype analyses, any misclassification within this category could not have biased the direct ischemic-versus-hemorrhagic mortality contrast, which was restricted to the more specific and better-validated I63 and I60–I62 code groups; within the overall comparison, the inclusion of these genuine cerebrovascular events would be expected to bias the stroke-associated estimate toward the null rather than to exaggerate it. More broadly, administrative coding variability across institutions introduces potential misclassification bias, which is an inherent limitation of retrospective database studies. The absence of granular clinical data limits the mechanistic interpretation of subtype-specific outcome differences. In addition, stroke was ascertained only within the 24 h peri-cannulation window; this definition was chosen to preserve a plausible temporal link between cannulation and neurologic injury, but it does not capture strokes occurring later during ECMO support or weaning, and the reported frequencies therefore reflect early peri-cannulation events rather than the cumulative incidence of stroke across the entire course of support.

Third, seizure diagnoses were ascertained by ICD-10 codes without electroencephalographic confirmation, likely underrepresenting true seizure burden, particularly in sedated and mechanically ventilated patients. Palliative care utilization and hospital readmissions may similarly be subject to inconsistent coding practices across institutions. Mortality was ascertained as all-cause mortality, and the contribution of neurologic death or withdrawal of life-sustaining therapy cannot be determined from administrative data alone; consequently, cause-specific mortality, including the distinction between neurological and non-neurological death, could not be characterized overall or within the stroke and no-stroke subgroups.

Finally, while TriNetX aggregates data from healthcare organizations across multiple sites, the precise geographic composition of the analytic cohort could not be defined, which may limit generalizability across healthcare systems with differing ECMO practices, anticoagulation protocols, and neurologic monitoring standards, particularly outside the United States.

## 5. Conclusions

Stroke occurring within 24 h of ECMO cannulation is associated with substantially worse short- and long-term survival, with hemorrhagic subtype and markers of neurologic injury severity carrying the greatest prognostic burden. Hemorrhagic stroke was the only subtype independently associated with increased 30-day mortality in multivariable modeling, with more than half of affected patients dying within 30 days. Beyond mortality, stroke was associated with markedly higher rates of palliative care utilization and hospital readmission, outcomes that have received limited systematic attention in the prior literature but carry direct relevance to goals-of-care planning and resource allocation. These findings should be interpreted in light of the limitations inherent to this design; accordingly, the associations reported here are hypothesis-generating rather than causal. Within these constraints, our results are consistent with and extend prior ELSO registry evidence in an independent real-world EHR environment, and they support attention to early stroke subtype recognition, neurologic monitoring, and timely goals-of-care discussions in adult ECMO patients experiencing cerebrovascular complications, while underscoring the need for prospective studies incorporating granular physiologic and ECMO-specific data.

## Figures and Tables

**Table 1:** Baseline characteristics before and after propensity score matching for stroke vs. no stroke.

Characteristic	Stroke (*n* = 1481)	No Stroke (*n* = 17,500)	*p*-Value (Pre)	SMD (Pre)	Stroke (*n* = 1480)	No Stroke (*n* = 1480)	*p*-Value (Post)	SMD (Post)
Demographics								
Age, mean ± SD (year)	52.8 ± 16.1	51.7 ± 16.9	0.0112	0.0702	52.9 ± 16.0	53.3 ± 16.3	0.4418	0.0283
Male, *n* (%)	961 (64.9)	11,289 (64.5)	0.7691	0.008	960 (64.9)	968 (65.4)	0.7577	0.0113
Female, *n* (%)	520 (35.1)	6202 (35.4)	0.7996	0.0069	520 (35.1)	512 (34.6)	0.7577	0.0113
White	936 (63.2)	9953 (56.9)	<0.0001	0.1294	935 (63.2)	928 (62.7)	0.7899	0.0098
Black or African American	151 (10.2)	2518 (14.4)	<0.0001	0.128	151 (10.2)	146 (9.9)	0.7597	0.0112
Asian	44 (3.0)	516 (2.9)	0.961	0.0013	44 (3.0)	48 (3.2)	0.6718	0.0156
Hispanic or Latino	124 (8.4)	1608 (9.2)	0.2951	0.0288	124 (8.4)	123 (8.3)	0.947	0.0024
**Cardiac diagnoses**								
Heart failure	262 (17.7)	5633 (32.2)	<0.0001	0.340	262 (17.7)	248 (16.8)	0.4956	0.0251
Atrial fibrillation/flutter	153 (10.3)	3878 (22.2)	<0.0001	0.3249	153 (10.3)	149 (10.1)	0.8081	0.0089
Acute myocardial infarction	152 (10.3)	2640 (15.1)	<0.0001	0.1453	152 (10.3)	139 (9.4)	0.4222	0.0295
Cardiomyopathy	120 (8.1)	2509 (14.3)	<0.0001	0.1985	120 (8.1)	117 (7.9)	0.839	0.0075
Cardiac arrest	66 (4.5)	2168 (12.4)	<0.0001	0.2886	66 (4.5)	73 (4.9)	0.5431	0.0224
**Pulmonary diagnoses**								
Respiratory failure	259 (17.5)	7232 (41.3)	<0.0001	0.5421	259 (17.5)	248 (16.8)	0.5915	0.0197
Pneumonia	135 (9.1)	3460 (19.8)	<0.0001	0.3067	135 (9.1)	114 (7.7)	0.1643	0.0511
Pulmonary embolism	52 (3.5)	1290 (7.4)	<0.0001	0.1708	52 (3.5)	64 (4.3)	0.2557	0.0418
**Neurologic/Muscular**								
Unspecified convulsions	35 (2.4)	780 (4.5)	0.0001	0.1156	34 (2.3)	36 (2.4)	0.8088	0.0089
Epilepsy/seizures	27 (1.8)	566 (3.2)	0.0027	0.09	27 (1.8)	22 (1.5)	0.4714	0.0265
Anoxic brain damage	20 (1.4)	406 (2.3)	0.0156	0.0723	20 (1.4)	28 (1.9)	0.2444	0.0428
**Other conditions**								
Acute kidney failure	223 (15.1)	6371 (36.4)	<0.0001	0.5036	223 (15.1)	227 (15.3)	0.8378	0.0075
Shock, unspecified	171 (11.5)	5448 (31.1)	<0.0001	0.4923	171 (11.5)	183 (12.4)	0.4967	0.025
Other sepsis	132 (8.9)	3895 (22.3)	<0.0001	0.3743	132 (8.9)	132 (8.9)	1.00	<0.0001
GI hemorrhage	31 (2.1)	1060 (6.1)	<0.0001	0.2015	31 (2.1)	35 (2.4)	0.6185	0.0183
**ECMO modality**								
VA-ECMO, peripheral	1077 (72.7)	10,990 (62.8)	<0.0001	0.2132	1076 (72.7)	1068 (72.2)	0.7734	0.0118
VV-ECMO, peripheral	457 (30.9)	7814 (44.7)	<0.0001	0.2871	457 (30.9)	461 (31.1)	0.9051	0.0046
**Medications**								
Anticoagulants	529 (35.7)	10,272 (58.7)	<0.0001	0.473	529 (35.7)	512 (34.6)	0.5129	0.0241

Note: Values are reported as mean ± SD or n (%). Patients may receive more than one ECMO modality during the same hospitalization; therefore, modality category percentages may exceed 100%. SMD, standardized mean difference; VA-ECMO, venoarterial ECMO; VV-ECMO, venovenous ECMO; GI, gastrointestinal.

**Table 2:** Multivariable Cox Proportional Hazards Model for 30-Day Mortality in ECMO Patients with Stroke vs. No Stroke.

Covariate	HR	95% CI	*p*
ECMO with stroke vs. without stroke	1.368	1.209–1.548	<0.0001
Male sex	1.055	0.999–1.113	0.0536
Age at index (per year)	1.021	1.019–1.022	<0.0001
Hypertension	0.895	0.849–0.944	<0.0001
Paroxysmal tachycardia	0.892	0.837–0.950	0.0004
Atrial fibrillation/flutter	0.833	0.785–0.885	<0.0001
Other cardiac arrhythmias	0.884	0.832–0.938	<0.0001
Heart failure	0.988	0.927–1.052	0.7072
Cardiomyopathy	0.865	0.806–0.927	<0.0001
Seizure disorder	0.962	0.859–1.078	0.5073
Cardiac arrest	1.400	1.321–1.483	<0.0001
Shock	1.318	1.236–1.405	<0.0001
Septicemia	0.930	0.878–0.985	0.0129
Acute kidney injury	1.415	1.326–1.511	<0.0001
Gastrointestinal hemorrhage	0.945	0.864–1.033	0.2134
Coma	1.285	1.160–1.423	<0.0001
NIHSS score 0–9	0.373	0.167–0.835	0.0165
NIHSS score 10–19	0.582	0.311–1.091	0.0913
NIHSS score 20–29	0.734	0.469–1.150	0.1766
Cerebral compression/herniation	2.198	1.857–2.601	<0.0001
Cerebral edema	2.274	1.986–2.603	<0.0001
VA-ECMO	1.501	1.378–1.636	<0.0001
VV-ECMO	0.857	0.801–0.916	<0.0001

Note: Multivariable Cox proportional hazards model adjusted for demographic characteristics, comorbidities, neurologic severity, and ECMO modality. HRs are reported with 95% confidence intervals. HR, hazard ratio; CI, confidence interval; NIHSS, National Institutes of Health Stroke Scale.

## Data Availability

Restrictions apply to the availability of these data. Data were obtained from the TriNetX Research Network under license and are not publicly available. Access may be granted to qualified researchers from the authors with the permission of TriNetX, subject to TriNetX policies and institutional agreements.

## Supplementary Materials

The following supporting information can be downloaded at: https://www.mdpi.com/article/10.3390/jcm15124790/s1, Table S1: ICD-10 and ICD-10-PCS code definitions for exposures, covariates, and outcomes; Table S2: Study Population Demographics (Total ECMO Cohort, 2015–2025); Table S3: Clinical outcomes after propensity score matching in ECMO patients with and without stroke; Table S4: Clinical outcomes after propensity score matching in ECMO patients with ischemic vs. hemorrhagic stroke; Table S5: Multivariable Cox Proportional Hazards Model for 30-Day Mortality in ECMO Patients with Ischemic Stroke vs. No Stroke; Table S6: Multivariable Cox Proportional Hazards Model for 30-Day Mortality in ECMO Patients with Hemorrhagic Stroke vs. No Stroke; Table S7: Multivariable Cox Proportional Hazards Model for 30-Day Mortality Comparing Ischemic vs. Hemorrhagic Stroke.

## Abbreviations

The following abbreviations are used in this manuscript:

AIS	acute ischemic stroke
AHA	American Heart Association
CI	confidence interval
ECMO	extracorporeal membrane oxygenation
ECPR	extracorporeal cardiopulmonary resuscitation
EEG	electroencephalography
EHR	electronic health record
ELSO	Extracorporeal Life Support Organization
HR	hazard ratio
HS	hemorrhagic stroke
ICD-10	International Classification of Diseases, 10th Revision
OR	odds ratio
PSM	propensity score matching
RR	risk ratio
SMD	standardized mean difference
STROBE	Strengthening the Reporting of Observational Studies in Epidemiology
VA	venoarterial
VV	venovenous
