# Supplementary material for: Stroke Subtype as a Determinant of Mortality in Adult Patients on Extracorporeal Membrane Oxygenation
Source: J Clin Med. 2026 Jun 20;15(12):4790. doi: 10.3390/jcm15124790 (PMC13302652; doi:10.3390/jcm15124790)
Supplement: Supplementary file 1 [file jcm-15-04790-s001.zip › jcm-4362508-supplementary.pdf]

Supplementary Materials

Stroke Subtype as a Determinant of Mortality in Adult Patients on Extracorporeal Membrane Oxygenation

Table S1. ICD-10 and ICD-10-PCS code definitions for exposures, covariates, neurologic injury severity, and outcomes used in this study.

Domain	Variable	Code(s)
Exposure	Any stroke	I60–I63
	Acute ischemic stroke	I63
	Hemorrhagic stroke	I60–I61
	Unspecified cerebrovascular events	I64
Neurologic injury severity	Cerebral edema	G93.6
	Cerebral compression/herniation	G93.5
	Coma	R40.20–R40.24
	Anoxic brain damage	G93.1
	Epilepsy/recurrent seizures	G40
	Unspecified convulsions	R56.9
Neuromuscular	Critical illness myopathy	G72.81
	Critical illness polyneuropathy	G62.81
Cardiac diagnoses	Heart failure	I50
	Atrial fibrillation/flutter	I48
	Other cardiac arrhythmias	I49
	Paroxysmal tachycardia	I47
	Cardiomyopathy	I42
	Acute myocardial infarction	I21
	Subsequent STEMI/NSTEMI	I22
	Acute myocarditis	I40
	Myocarditis, unspecified	I51.4
Outcomes	Cardiac arrest	I46
	Respiratory failure	J96
	Pneumonia, unspecified	J18
	Bacterial pneumonia	J15
	Viral pneumonia	J12
	Pneumonia (S. pneumoniae)	J13
	Pneumonia (H. influenzae)	J14
	Pulmonary embolism	I26
Other systemic	Acute kidney failure	N17
	Shock, not elsewhere classified	R57
	Sepsis (other/unspecified)	A41
	Other venous embolism/thrombosis	I82
	Gastrointestinal hemorrhage, unspecified	K92.2
Medication exposure	Anticoagulant use	BL110
ECMO characteristics	VA-ECMO	5A1522G, 5A15A2G
	VV-ECMO	5A1522H, 5A15A2H

Utilization outcomes	Hospital readmission	32485007 (SNOMED CT)
	Palliative care encounter	Z51.5

Note: ICD-10 = International Classification of Diseases, 10th Revision; ICD-10-PCS = Procedure Coding System; SNOMED CT = Systematized Nomenclature of Medicine — Clinical Terms.

Table S2. Study Population Demographics (Total ECMO Cohort, 2015–2025).

Characteristic	Value (Stroke vs. No Stroke)	Source
Study period	2015–2025	TriNetX US Collaborative Network
Age, mean ± SD (yr)	Stroke: 52.8 ± 16.1; No Stroke: 51.7 ± 16.9	Pre-PSM
Male, n (%)	Stroke: 961 (64.9); No Stroke: 11,289 (64.5)	Pre-PSM
Female, n (%)	Stroke: 520 (35.1); No Stroke: 6,202 (35.4)	Pre-PSM
White, n (%)	Stroke: 936 (63.2); No Stroke: 9,953 (56.9)	Pre-PSM
Black or African American, n (%)	Stroke: 151 (10.2); No Stroke: 2,518 (14.4)	Pre-PSM
Hispanic or Latino, n (%)	Stroke: 124 (8.4); No Stroke: 1,608 (9.2)	Pre-PSM
Asian, n (%)	Stroke: 44 (3.0); No Stroke: 516 (2.9)	Pre-PSM
Any stroke	1,481 (7.8%)	Pre-PSM
No stroke	17,500 (92.2%)	Pre-PSM
Matched stroke	1,480	Post-1:1 PSM
Matched no stroke	1,480	Post-1:1 PSM
Anticoagulant use (BL110)	Stroke: 529 (35.7); No Stroke: 10,272 (58.7)	Pre-PSM

Note: PSM, propensity score matching. Demographic categories are non-mutually exclusive for race/ethnicity due to TriNetX classification.

Table S3. Clinical outcomes after propensity score matching in ECMO patients with and without stroke (30 days, 90 days, 1 year).

Outcome	Time	Stroke (n=1,480)	No stroke (n=1,480)	RR (95% CI)	p
All-cause mortality	30 d	672 (45.4)	580 (39.2)	1.16 (1.07–1.26)	0.0006
All-cause mortality	90 d	778 (52.6)	662 (44.7)	1.18 (1.09–1.27)	<0.0001
All-cause mortality	1 yr	844 (57.0)	713 (48.2)	1.18 (1.11–1.27)	<0.0001
Cardiac arrest	30 d	615 (41.6)	392 (26.5)	1.57 (1.41–1.74)	<0.0001
Cardiac arrest	90 d	627 (42.4)	406 (27.4)	1.54 (1.40–1.71)	<0.0001
Cardiac arrest	1 yr	633 (42.8)	410 (27.7)	1.54 (1.40–1.71)	<0.0001
Epilepsy/recurrent seizures	30 d	121 (8.2)	57 (3.9)	2.12 (1.56–2.88)	<0.0001
Epilepsy/recurrent seizures	90 d	131 (8.9)	61 (4.1)	2.15 (1.60–2.89)	<0.0001
Epilepsy/recurrent seizures	1 yr	146 (9.9)	66 (4.5)	2.21 (1.67–2.93)	<0.0001
Palliative care encounter*	30 d	644 (44.9)	351 (24.9)	1.80 (1.62–2.01)	<0.0001
Palliative care encounter*	90 d	664/1,435 (46.3)	362/1,410 (25.7)	1.80 (1.62–2.00)	<0.0001
Palliative care encounter*	1 yr	675/1,435 (47.0)	373/1,410 (26.5)	1.78 (1.60–1.97)	<0.0001
Hospital readmission	30 d	831 (56.1)	590 (39.9)	1.41 (1.30–1.52)	<0.0001
Hospital readmission	90 d	871 (58.9)	609 (41.1)	1.43 (1.33–1.54)	<0.0001

Hospital readmission	1 yr	883 (59.7)	628 (42.4)	1.41 (1.31–1.51)	<0.0001

*Note: Values are reported as n (%) unless otherwise specified. *Palliative care encounter denominators differ due to data availability (events / available patients). RR, risk ratio; CI, confidence interval.*

Table S4. Clinical outcomes after propensity score matching in ECMO patients with ischemic vs. hemorrhagic stroke (30 days, 90 days, 1 year).

Outcome	Time	AIS, n (%)	HS, n (%)	Risk Difference (95% CI)	RR (95% CI)	OR (95% CI)	p
All-cause mortality	30 d	146 (36.05)	228 (56.30)	−20.25 (−26.97 to −13.52)	0.64 (0.55–0.75)	0.44 (0.33–0.58)	<0.0001
All-cause mortality	90 d	175 (43.21)	261 (64.44)	−21.24 (−27.94 to −14.53)	0.67 (0.59–0.77)	0.42 (0.32–0.56)	<0.0001
All-cause mortality	1 yr	199 (49.14)	274 (67.65)	−18.52 (−25.19 to −11.85)	0.73 (0.64–0.82)	0.46 (0.35–0.61)	<0.0001
Cardiac arrest	30 d	180 (44.44)	159 (39.26)	5.19 (−1.60 to 11.97)	1.13 (0.96–1.33)	1.24 (0.94–1.64)	0.1347
Cardiac arrest	90 d	187 (46.17)	159 (39.26)	6.91 (0.12 to 13.71)	1.18 (1.00–1.38)	1.33 (1.00–1.76)	0.0467
Cardiac arrest	1 yr	187 (46.17)	161 (39.75)	6.42 (−0.38 to 13.22)	1.16 (0.99–1.36)	1.30 (0.98–1.72)	0.065
Epilepsy/recurrent seizures	30 d	26 (6.42)	27 (6.67)	−0.25 (−3.65 to 3.16)	0.96 (0.57–1.62)	0.96 (0.55–1.68)	0.887
Epilepsy/recurrent seizures	90 d	29 (7.16)	30 (7.41)	−0.25 (−3.83 to 3.33)	0.97 (0.59–1.58)	0.96 (0.57–1.64)	0.8924
Epilepsy/recurrent seizures	1 yr	35 (8.64)	33 (8.15)	0.49 (−3.33 to 4.31)	1.06 (0.67–1.67)	1.07 (0.65–1.75)	0.8
Palliative care encounter*	30 d	169/395 (42.79)	184/396 (46.47)	−3.68 (−10.60 to 3.24)	0.92 (0.79–1.08)	0.86 (0.65–1.14)	0.2979
Palliative care encounter*	90 d	176/395 (44.56)	187/396 (47.22)	−2.67 (−9.61 to 4.28)	0.94 (0.81–1.10)	0.90 (0.68–1.19)	0.452
Palliative care encounter*	1 yr	178/395 (45.06)	190/396 (47.98)	−2.92 (−9.87 to 4.03)	0.94 (0.81–1.09)	0.89 (0.67–1.18)	0.4109
Hospital readmission	30 d	221 (54.57)	200 (49.38)	5.19 (−1.69 to 12.06)	1.11 (0.97–1.26)	1.23 (0.93–1.62)	0.1397
Hospital readmission	90 d	235 (58.03)	209 (51.61)	6.42 (−0.42 to 13.26)	1.12 (0.99–1.28)	1.30 (0.98–1.71)	0.0664
Hospital readmission	1 yr	239 (59.01)	211 (52.10)	6.91 (0.09 to 13.74)	1.13 (1.00–1.28)	1.32 (1.00–1.75)	0.0477

*Note: Risk Difference reported as percentage points. *Palliative care encounter denominators differ due to data availability. AIS, acute ischemic stroke; HS, hemorrhagic stroke; RR, risk ratio; OR, odds ratio; CI, confidence interval.*

Table S5. Multivariable Cox Proportional Hazards Model for 30-Day Mortality in ECMO Patients with Ischemic Stroke Versus No Stroke.

Covariate	HR	β	SE	z	p	95% CI
Ischemic stroke (vs. no stroke)	0.985	–0.015	0.063	–0.235	0.8138	0.870–1.116
Male sex	1.042	0.041	0.031	1.354	0.1756	0.982–1.106
Age at index (per year)	1.018	0.018	0.001	18.456	<0.0001	1.017–1.020
Hypertension	0.828	–0.189	0.034	–5.530	<0.0001	0.774–0.885
Paroxysmal tachycardia	0.890	–0.117	0.041	–2.843	0.0045	0.821–0.964
Atrial fibrillation/flutter	0.867	–0.143	0.038	–3.756	0.0002	0.805–0.934

Other cardiac arrhythmias	0.863	–0.147	0.040	–3.727	0.0002	0.799–0.933
Heart failure	0.927	–0.076	0.037	–2.053	0.0401	0.862–0.997
Cardiomyopathy	0.815	–0.204	0.049	–4.199	<0.0001	0.741–0.897
Seizure disorder	1.041	0.040	0.075	0.539	0.5901	0.900–1.205
Cardiac arrest	1.365	0.311	0.036	8.706	<0.0001	1.273–1.464
Shock	1.288	0.253	0.036	6.978	<0.0001	1.199–1.382
Sepsis	0.924	–0.079	0.037	–2.123	0.0337	0.859–0.994
Acute kidney injury	1.377	0.320	0.037	8.711	<0.0001	1.282–1.480
Gastrointestinal hemorrhage	0.948	–0.054	0.067	–0.807	0.4197	0.832–1.080
Coma	1.210	0.191	0.068	2.809	0.005	1.059–1.382
NIHSS score 0–9	0.129	–2.046	1.002	–2.043	0.0411	0.018–0.920
NIHSS score 10–19	0.330	–1.110	0.580	–1.914	0.0556	0.106–1.027
NIHSS score 20–29	0.364	–1.012	0.381	–2.654	0.008	0.172–0.768
Brain compression	2.405	0.878	0.123	7.125	<0.0001	1.889–3.062
Cerebral edema	2.595	0.954	0.094	10.177	<0.0001	2.160–3.118
VA-ECMO	1.577	0.456	0.048	9.589	<0.0001	1.437–1.731

Note: HR, hazard ratio; SE, standard error; CI, confidence interval; NIHSS, National Institutes of Health Stroke Scale; VA-ECMO, venoarterial ECMO.

Table S6. Multivariable Cox Proportional Hazards Model for 30-Day Mortality in ECMO Patients with Hemorrhagic Stroke Versus No Stroke.

**Covariate**	**Hazard ratio**	**β**	**SE**	**z**	**p**	**95% CI**
Hemorrhagic stroke (vs no stroke)	1.374	0.318	0.072	4.44	< 0.0001	1.194–1.581
Male sex	1.052	0.05	0.029	1.73	0.0836	0.993–1.113
Age at index (per year)	1.018	0.018	0.001	19.032	< 0.0001	1.016–1.020
Hypertension	0.833	−0.183	0.032	−5.655	< 0.0001	0.782–0.887
Paroxysmal tachycardia	0.883	−0.125	0.038	−3.244	0.0012	0.819–0.952
Atrial fibrillation or flutter	0.869	−0.140	0.036	−3.936	< 0.0001	0.810–0.932
Other cardiac arrhythmias	0.912	−0.092	0.037	−2.478	0.0132	0.848–0.981
Heart failure	0.917	−0.086	0.035	−2.456	0.0141	0.857–0.983
Cardiomyopathy	0.798	−0.226	0.045	−5.507	< 0.0001	0.730–0.871
Seizure disorder	0.983	−0.017	0.075	−0.223	0.8237	0.849–1.139
Cardiac arrest	1.434	0.36	0.034	10.752	< 0.0001	1.343–1.531
Shock	1.282	0.249	0.035	7.163	< 0.0001	1.198–1.373
Sepsis	0.937	−0.065	0.035	−1.848	0.0647	0.875–1.004
Acute kidney injury	1.383	0.325	0.035	9.335	< 0.0001	1.292–1.481

Gastrointestinal hemorrhage	0.942	−0.060	0.061	−0.971	0.3317	0.835–1.063
Coma	1.235	0.211	0.065	3.253	0.0011	1.087–1.402
NIHSS score 0–9	< 0.0001	−11.708	255.888	−0.046	0.9635	Not estimable
NIHSS score 10–19	0.293	−1.227	1.002	−1.225	0.2207	0.041–2.089
NIHSS score 20–29	0.387	−0.949	0.709	−1.340	0.1803	0.097–1.552
Brain compression	2.186	0.782	0.105	7.432	< 0.0001	1.779–2.687
Cerebral edema	2.3	0.833	0.084	9.923	< 0.0001	1.951–2.712
VA-ECMO, central	1.561	0.445	0.047	9.553	< 0.0001	1.425–1.711
VV-ECMO, peripheral	0.877	−0.132	0.036	−3.643	0.0003	0.817–0.941
Anticoagulant use	1.082	0.079	0.083	0.944	0.345	0.919–1.273
Any antithrombotic agent	0.837	−0.178	0.092	−1.941	0.0519	0.700–1.001
Antiplatelet therapy	1.139	0.13	0.032	4.013	< 0.0001	1.069–1.213

Table S7. Multivariable Cox Proportional Hazards Model for 30-Day Mortality Comparing Ischemic Versus Hemorrhagic Stroke.

**Covariate**	**Hazard ratio**	**β**	**SE**	**z**	**p**	**95% CI**
Stroke subtype (Ischemic vs Hemorrhagic)	0.685	–0.379	0.094	–4.044	< 0.0001	0.570–0.823
Male sex	1.071	0.069	0.092	0.752	0.4523	0.895–1.282
Age at index (per year)	1.016	0.016	0.003	5.186	< 0.0001	1.010–1.022
Hypertension	0.852	–0.160	0.095	–1.688	0.0915	0.707–1.026
Paroxysmal tachycardia	0.828	–0.188	0.112	–1.686	0.0919	0.666–1.031
Atrial fibrillation or flutter	0.788	–0.238	0.105	–2.276	0.0229	0.642–0.967
Other cardiac arrhythmias	0.999	–0.001	0.11	–0.005	0.9962	0.806–1.240
Heart failure	0.787	–0.240	0.108	–2.214	0.0268	0.637–0.973
Cardiomyopathy	0.706	–0.348	0.141	–2.465	0.0137	0.535–0.931
Seizure disorder	1.384	0.325	0.164	1.983	0.0473	1.004–1.908
Cardiac arrest	1.093	0.089	0.099	0.889	0.3692	0.900–1.327
Shock	1.086	0.082	0.11	0.749	0.4537	0.875–1.347
Sepsis	0.661	–0.414	0.105	–3.948	< 0.0001	0.538–0.812

Acute kidney injury	1.374	0.318	0.109	2.926	0.0034	1.111–1.699
Gastrointestinal hemorrhage	0.96	–0.041	0.161	–0.252	0.8013	0.700–1.314
Coma	1.153	0.142	0.133	1.072	0.2836	0.889–1.496
NIHSS score 0–9	0.111	–2.195	1.003	–2.188	0.0287	0.016–0.796
NIHSS score 10–19	0.366	–1.004	0.507	–1.980	0.0477	0.136–0.990
NIHSS score 20–29	0.361	–0.510	0.324	–1.574	0.1154	0.318–1.133
Brain compression	2.47	0.906	0.127	6.189	< 0.0001	1.855–3.288
Cerebral edema	1.789	0.582	0.134	4.353	< 0.0001	1.377–2.324
VA-ECMO	1.186	0.171	0.167	1.019	0.3084	0.854–1.646
VV-ECMO	0.783	–0.244	0.129	–1.898	0.0577	0.609–1.008
Anticoagulant use	1.701	0.531	0.289	1.836	0.0663	0.965–2.999
Any antithrombotic agent	0.615	–0.486	0.309	–1.572	0.116	0.336–1.127
Antiplatelet therapy	1.005	0.005	0.105	0.05	0.9616	0.818–1.235
